# Prediction of on-water performance in outrigger canoeing using laboratory tests: the influence of sex and anthropometric characteristics

**DOI:** 10.3389/fspor.2026.1807781

**Published:** 2026-03-25

**Authors:** Filipe Estácio Costa, Rafael Lima Kons, Fabrizio Caputo, Juliano Dal Pupo

**Affiliations:** 1 Center of Sports, Federal University of Santa Catarina, Florianópolis, Santa Catarina, Brazil; 2 Department of Physical Education, Faculty of Education, Federal University of Bahia, Salvador, Bahia, Brazil; 3 College of Health and Sport Sciences, Human Performance Research Group, Santa Catarina State University, Florianópolis, Santa Catarina, Brazil

**Keywords:** allometric scaling, athletic performance, endurance exercise, field-based performance, power output

## Abstract

The relationship between laboratory-based ergometer performance, body size, and on-water outrigger canoe (OC) racing remains unclear. This study examined associations between anthropometric characteristics, ergometer-derived performance, and on-water endurance performance in OC athletes using a sex-specific approach. Competitive paddlers (22 males and 16 females) completed anthropometric assessments, an incremental test, a 1000-m time trial, and a maximum paddling power test on an OC-adapted ergometer, as well as a 4.18-km simulated on-water race. Sex-specific statistical analyses, including allometric scaling and regression models, were used to identify determinants of on-water performance. Male athletes exhibited greater body mass, height, lean body mass, and superior performance in all ergometer and on-water tests compared with females (*p* < 0.01). In females only, body mass and lean body mass were moderately to strongly associated with ergometer-derived power outputs (r = 0.66–0.85, *p* < 0.01), whereas no such relationships were observed in males. Significant allometric scaling effects were identified exclusively in females for power-based ergometer variables, indicating disproportionate increases in power with increasing body and lean mass. No anthropometric variables were associated with on-water performance in either sex. In regression analyses, incremental peak power was the strongest predictor of on-water performance in males (adjusted R^2^ = 0.69), whereas in females, 1000-m mean power allometrically adjusted for body mass best predicted on-water speed (adjusted R^2^ = 0.67). These findings emphasize the importance of sex-specific interpretation of ergometer assessments and consideration of body size when relating laboratory performance to real-world OC racing.

## 1 Introduction

The outrigger canoe (OC) has become a popular sport around the globe in the last decade. Although there is evidence of competition from approximately 100 years ago, the sport gained international attention following its inclusion in the Rio 2016 Paralympic Games. However, despite its growing popularity, there is still limited scientific literature on this sport, with most research emphasizing sprint performance and relying on ergometer-based assessments rather than on-water performance (1). The outrigger canoe has particular characteristics differing from traditional canoes and kayaks, as it features a main hull (canoe) connected to an outrigger float (ama) via two lateral supports (iakos), a structure that increases drag but enhances stability in rough water.

The OC performance depends on a complex interaction of anthropometric, physiological, biomechanical, and technical factors (1–3). Previous research in canoeing, kayaking, and rowing has identified parameters such as aerobic and anaerobic power, muscular strength, body composition, and stroke efficiency as key predictors of performance (4–6). While a significant amount of research has focused on sprint distances ranging from 200 to 1000 m in kayaking and OC (1, 2, 7, 8), endurance paddle disciplines such as long-distance OC races have received limited scientific attention, despite their popularity and distinct physiological demands. The International Va'a Federation (IVF) organizes biennial World Distance Championships and World Elite and Club Sprint Championships, while more recently the inclusion of OC in the International Canoe Federation's official 2026 long-distance ocean racing calendar reflects the sport's continued global growth and consolidation (9). Endurance events typically require different physiological and biomechanical adaptations compared to sprint disciplines due to longer race durations and distinct energy system contributions (10). In OC, even the shortest event in the individual category (straight 500-m) presents race times of 122 and 141 s for elite male and female athletes, respectively, indicating a substantial aerobic contribution (11).

The challenges associated with conducting controlled assessments in aquatic environments, such as variable weather conditions, water currents, and technical differences between boats, have led to the widespread use of ergometers in both training and research (2, 6, 12–16). Ergometer testing enables the reliable measurement of physiological and mechanical variables under standardized conditions, thereby facilitating performance monitoring and the evaluation of training adaptations (6). The 2000-m time trial, for instance, is a well-established indicator of rowing performance (6, 16), whereas shorter tests such as 1-min, 200-, 500-, or 1000-m time trials have been proposed for OC, slalom canoeing, dragon boat, and kayaking (2, 17–20). However, it remains unclear which ergometer tests best represent on-water performance in OC.

Different ergometer protocols may provide complementary insights into performance capacity (21). Incremental tests are commonly used to assess aerobic fitness and other physiological parameters (5, 12, 19, 22, 23). On the other hand, short maximal sprints or fixed-distance time trials (e.g., 15 maximum strokes, all-out 120-s or 1000-m tests) can assess the athlete's ability to generate and sustain high power outputs, integrating both aerobic and anaerobic contributions (2, 7, 24, 25). Understanding the relationship between these different test outcomes may help identify the most practical and valid methods for assessing physical performance potential in OC paddlers.

Body size and composition are additional determinants of paddling performance. While greater body mass may allow for higher absolute power generation on an ergometer, excess mass can negatively affect on-water performance by increasing drag (26, 27). Consequently, scaling performance variables to body mass or lean mass can provide a more accurate representation of athletic capacity (5, 14). Previous research in rowing suggests that including body mass in allometric models improves the prediction of on-water performance compared to ergometer results alone (28, 29). However, such relationships have not yet been established for outrigger canoe paddlers and all the studies that presented data about ergometer assessments used absolute power (W), time (s), stroke rate (s.min^−1^), and speed (m.s^−1^) to indicate performance, not taking into account any anthropometric characteristics (20, 24, 30).

Despite the increasing use of ergometers for training and testing in outrigger canoeing, the extent to which laboratory-based assessments reflect real on-water performance remains unclear. Moreover, the potential influence of anthropometric characteristics on these relationships has not been examined in this population. Therefore, the purpose of this study was to investigate the relationships between performance in different ergometer tests (incremental test, 1000-m time trial, and maximum paddling power), anthropometric variables (body mass, body fat percentage, and height) and their relationships with on-water performance during a 4.18-km OC race simulation in male and female athletes.

## 2 Materials and methods

### 2.1 Participants

Thirty-eight paddling athletes with experience in OC competition agreed to participate in the present study, including twenty-two males (age: 40.91 ± 11.30 years, OC experience: 4.90 ± 3.14 years, training hours/week: 6.18 ± 2.94 h) and sixteen females (age: 42.81 ± 7.66 years, OC experience: 4.83 ± 2.27 years, training hours/week: 6.59 ± 3.61 h). All athletes regularly participated in regional and national competitions during the competitive season. According to the athlete classification framework proposed by McKay et al. (31), most participants would be categorized as Tier 2 (“trained/developmental”), reflecting competitive but non-elite status. The inclusion criteria were: being at least 18 years old; reporting no injuries during the data collection period; having at least six months of regular training with a minimum frequency of three sessions per week; and having experience with on-water training using a V1R (OC1) boat. Prior to testing, participants were informed about the objectives of the study, as well as its risks and benefits, and provided written informed consent. This study was approved by the ethics committee of the university where all tests were performed. All procedures were conducted in accordance with the Declaration of Helsinki.

### 2.2 Study design and procedures

This is a descriptive cross-sectional study. Participants took part in five assessment sessions, with at least 24 h of rest between sessions. On the first visit, participants completed a questionnaire to characterize the sample and training history. Anthropometric measurements and body composition assessments were also performed. Participants then familiarized themselves with the adapted ergometer equipped with an OC attachment, which was used in the subsequent testing sessions.

On the second day, participants performed a maximal incremental test on the adapted ergometer to determine their maximal aerobic power. On the third day, participants completed a 1000-m time trial on the ergometer. On the fourth day, participants performed a maximum paddling power test on the ergometer. Finally, on the fifth day, participants completed a simulated on-water competition over a standardized course to assess on-water endurance performance. The testing protocols are described below.

#### 2.2.1 Anthropometric measurements

Body mass (kg) and height (cm) were measured using a digital scale and a stadiometer, respectively. Sitting height was measured in centimeters using a stadiometer with 1 mm resolution and a 50 cm wooden box. Participants sat on the ischial tuberosities, held their breath at full inspiration (inspiratory apnea), and kept the head aligned with the Frankfurt plane. The final value corresponded to the vertical distance from the top of the box to the vertex of the head.

Arm span was measured using a tape measure with 1 mm resolution affixed to the wall. Participants stood facing the wall with the shoulders abducted to 90°. With elbows fully extended and palms facing upward (supinated grip), participants placed the tip of the left middle finger at the 0 cm mark on the tape. The final measurement was defined as the distance between the 0 cm point (left middle finger) and the point corresponding to the tip of the right middle finger.

The whole-body scans were performed using dual-energy x-ray absorptiometry (DXA) (QDR-1500; Hologic Discovery A, Waltham, MA) to assess lean body mass, fat mass, and body fat percentage (%). Participants were positioned supine on the scanning bed with both arms pronated at their sides. To ensure consistent and reproducible positioning, the same DXA operator manually assisted all participants by aligning the head, torso, and pelvis; internally rotating and fixating the legs and feet at 45°; positioning the arms alongside the body within the DXA scanning zone.

#### 2.2.2 Incremental test on adapted ergometer

An incremental step test was performed using a Concept2 Model E ergometer (Concept2, Morrisville, Vermont, USA) equipped with an outrigger canoe paddling adapter developed by Paddle Sport Training Systems (Vermont Waterways, East Hardwick, Canada). The drag factor of the ergometer was set at 120 for all participants. Participants first completed a 4-min warm-up at a constant power output of 25 W, followed immediately by the first 2-min stage at the same workload (25 W), totaling 6 min at this initial intensity. Thereafter, power output increased by 15 W every 2 min, similar to that described by Kerr et al. (12). Participants were instructed to regulate their power output using real-time feedback from the ergometer display, which provides stroke-by-stroke power data, allowing them to adjust their effort in order to match the target power for each stage. Stroke rate was self-selected and not standardized. Verbal encouragement was provided throughout the test to motivate participants to reach maximum effort and to inform them when their power output deviated from the prescribed target for each stage.

The test was terminated when participants were unable to maintain the prescribed power output for five consecutive strokes or upon voluntary exhaustion. Participants were allowed to switch paddling sides at their discretion to simulate the natural paddling pattern used during on-water sessions. Maximum aerobic power (W) was defined as the power output achieved during the last fully completed stage. If the final stage was not completed, a proportional value was calculated based on the time completed within that stage.

#### 2.2.3 1000-m time trial performance

A 5-min warm-up was performed at a power corresponding to 50% of the maximum aerobic power reached in the incremental test using the same ergometer described above. Subsequently, a 2-min rest interval was given before starting the test. Participants were instructed to complete the 1000-m course in the shortest possible time, using their own strategy and pacing. The intensity was self-selected, as well as the stroke rate and paddling side changes. The time to complete the test (s) was recorded and used to calculate 1000-m speed. For analysis, the 1000-m speed (m.s^−1^) and the mean power (W) were considered the performance outcomes of the time trial.

#### 2.2.4 Maximum paddling power test

Maximum paddling power output was assessed during a test comprising 20 maximal strokes performed on the adapted ergometer. Participants started the testing protocol with a 5-min warm-up at a power corresponding to 50% of the maximum aerobic power reached in the incremental test, followed by 15 submaximal strokes on each side. A 2-min rest interval was given before the test. The test was first performed using only one side for paddling, in a randomized order, and after a 5-min rest, the same procedure was repeated for the opposite side. A 10-min rest interval was provided before repeating the protocol for a second attempt. Since it was not possible to export ergometer stroke-by-stroke data directly from the Concept2 software, a video camera (iPhone 14 Pro Max, 60 fps, Apple, USA) was positioned behind the paddler and focused on the ergometer display to capture the power output of each stroke. Power values were subsequently analyzed frame by frame. The maximum paddling power output (W) among the 20 strokes was selected for analysis for both the right and left sides. As no significant differences were observed between sides, the mean value of both sides was used for subsequent analyses.

#### 2.2.5 On-water performance

For the on-water performance testing, participants performed a 4.18-km race simulating a real competition. The course was set in a brackish water lagoon and a technical briefing was provided beforehand to explain the route and starting and finishing procedures. Athletes performed their warm-up routines freely, following their usual pre-competition habits. They were instructed to complete the course in the shortest possible time. Paddling intensity, stroke rate, and paddling side changes were all self-selected. Each participant used their own canoe and paddle according to personal preference.

For safety reasons, all participants were encouraged to wear a flotation aid vest and a leash attached to the canoe. Due to logistical constraints, the race was divided into two heats: the first heat was assigned to male participants and the second to female participants, both following the same course. Environmental conditions (e.g., wind, water movement, and tide) were not instrumentally monitored during testing. However, all athletes within each sex competed simultaneously in their respective heat, minimizing potential environmental variability between competitors. The total time (s) to complete the course was recorded using a stopwatch and confirmed with a photo finish. On-water speed (m.s^−1^) was then calculated and used as the primary performance variable for this test.

### 2.3 Statistical analysis

Descriptive statistics (mean and standard deviation) were calculated for all variables. Normality of the data was assessed using the Shapiro–Wilk test. Comparisons between male and female participants were performed using Student's t-test when data were normally distributed; otherwise, the Mann–Whitney test was applied. The assumption of homogeneity of variances was assessed using Levene's test, and when variances were unequal (*p* < 0.05), Welch's *t*-test was used. Effect sizes were calculated using Cohen's *d* with 95% confidence intervals to assess the magnitude of differences between sexes. Pearson's correlation coefficients were computed to examine the relationships between anthropometric variables and performance in different ergometer tests. The magnitude of correlations (r) was interpreted as follows: 0.00–0.30 negligible, 0.30–0.50 weak, 0.50–0.70 moderate, 0.70–0.90 strong, and > 0.90 very strong (32). To account for potential body size–related effects on performance, an allometric analysis was performed using log–log linear regression models. For each ergometer-derived performance variable (maximum paddling power, maximum aerobic power from the incremental test, and 1000-m mean power), the relationship between body mass (*M*) and performance (P) was examined according to the equation: log *P* = log a + b log M where a represents the intercept and b the allometric exponent describing how performance scales with body mass. This approach allows quantification of whether performance increases proportionally (*b* ≈ 1), less than proportionally (*b* < 1), or more than proportionally (*b* > 1) with increasing body mass. Separate regressions were conducted for males and females to examine potential sex-specific scaling effects. The statistical significance of the regression models and the exponent b were determined for each test. When a significant relationship between body mass and performance was observed, normalized (size-adjusted) values were calculated using the equation: P_adj_ = P/M^b^. This adjustment removes the influence of body size, allowing comparisons of performance independently of body mass. For variables in which body mass showed no significant relationship with performance, raw (non-adjusted) values were retained for subsequent analyses. Regression analyses were conducted using ordinary least squares, and model fit was expressed by the coefficient of determination (R^2^) and *p*-values for the exponent b. The allometric scaling approach was preferred over simple ratio normalization (e.g., W.kg^−1^) because it avoids the assumption of direct proportionality (*b* = 1) and provides a data-driven estimation of the scaling exponent specific to the sample and test condition (28, 33).

Finally, to examine the determinants of on-water paddling performance, linear regression analyses were conducted separately for males and females. Prior to selecting the final analytical approach, exploratory multiple regression models were tested within each sex. However, substantial multicollinearity was identified among ergometer-derived performance variables, as indicated by elevated variance inflation factors, resulting in unstable coefficient estimates. Therefore, simple linear regressions were performed to assess the individual contribution of each ergometer-derived power variable to on-water paddling speed, with each ergometer test outcome entered as a single predictor. For female participants, additional analyses were conducted using allometrically adjusted power values (P/M^b^), where b corresponded to the scaling exponents derived from separate regressions between body mass and each ergometer performance variable. All statistical analyses were performed using R (RStudio), and the level of significance was set at *p* < 0.05.

## 3 Results

Table 1 presents the descriptive data of anthropometric characteristics, on-water performance, and ergometer test performance, as well as comparisons between male and female paddlers. Significant differences were observed between males and females for both anthropometric variables and paddling performance measures. Male participants exhibited greater body mass, height, lean body mass, arm span, and sitting height, as well as a lower body fat percentage, compared with female participants. Regarding paddling performance, male athletes demonstrated higher mean speed and power output and shorter completion times compared with female athletes. Based on these differences, separate analyses were conducted to examine correlations between anthropometric variables and paddling performance by sex.

**Table 1:** Anthropometric characteristics, on-water performance, and ergometer test performance in male and female paddlers.

Variable	Males		Females			
	*n*	Mean ± SD	*n*	Mean ± SD	*p*-value	ES [CI 95%]
Anthropometric						
Body mass (kg)	22	82.4 ± 9.9	16	64.1 ± 8.0	<0.01	1.99 [1.20, 2.78]
Height (cm)	22	178.8 ± 7.3	16	166.3 ± 8.6	<0.01	1.58 [0.83, 2.31]
Body fat (%)	22	18.2 ± 4.1	16	28.3 ± 4.8	<0.01	−2.28 [−3.11, −1.44]
Lean body mass (kg)	22	62.84 ± 7.58	16	42.70 ± 5.16	<0.01	3.01 [2.06, 3.95]
Arm span (cm)	22	183.42 ± 7.53	16	167.44 ± 8.75	<0.01	1.98 [1.18, 2.76]
Sitting height (cm)^a^	22	94.84 ± 3.95	16	87.05 ± 7.29	<0.01	1.39 [0.67, 2.11]
On-water performance						
Completion time (s)	13	1746.4 ± 146.4	11	1944.7 ± 199.4	0.01	−1.15 [−2.01, −0.27]
Mean speed (m.s^−1^)	13	2.4 ± 0.2	11	2.2 ± 0.2	<0.01	1.20 [0.31, 2.07]
Ergometer test performance						
1000-m mean speed (m.s^−1^)	22	3.4 ± 0.2	16	2.7 ± 0.2	<0.01	3.26 [2.26, 4.24]
1000-m mean power (W)	22	106.7 ± 15.2	16	57.3 ± 14.5	<0.01	3.31 [2.30, 4.30]
1000-m mean stroke rate (spm)	22	54.05 ± 7.26	16	56.25 ± 5.26	0.31	−0.34 [−0.98, 0.31]
Maximal aerobic power (W)	22	107.9 ± 14.9	16	57.8 ± 12.4	<0.01	3.62 [2.55, 4.66]
Maximum paddling power (W)	21	208.1 ± 24	15	109.0 ± 26.4	<0.01	3.96 [2.81, 5.10]

spm, stroke per minute. Some *n* differences between tests are due to logistical and availability constraints.

^a^ Welch *t*-test.

Regarding the relationship between anthropometric measures and paddling performance, significant correlations were observed between total lean body mass and body mass with performance across all ergometer-based variables (*p* < 0.01), but only among female participants. Arm span also showed a significant correlation (*p* < 0.05) with performance in the maximum paddling power test, only in females. For males, none variables correlated significantly with performance in any test (Table 2).

**Table 2:** Pearson correlation coefficients (*r*) between anthropometric variables and paddling performance variables in ergometer and on-water tests, separated by sex.

Variable	Maximal aerobic power (W)	Maximum paddling power (W)	1000-m mean power (W)	1000-m mean speed (m.s^−1^)	On-water mean speed (m.s^−1^)
Males					
Body mass (kg)	0.08	0.17	0.33	0.33	−0.07
Body fat (%)	−0.24	−0.16	−0.06	−0.08	0.37
Lean body mass (kg)	0.24	0.32	0.40	0.41	−0.20
Height (cm)	0.04	0.09	0.32	0.31	0.15
Sitting height (cm)	0.13	0.29	0.39	0.37	0.06
Arm span (cm)	0.07	0.22	0.34	0.35	−0.05
Females					
Body mass (kg)	0.73*	0.72*	0.69*	0.66*	−0.05
Body fat (%)	−0.17	−0.24	−0.19	−0.23	−0.45
Lean body mass (kg)	0.85*	0.85*	0.83*	0.81*	0.17
Height (cm)	0.42	0.42	0.45	0.44	0.42
Sitting height (cm)	0.21	0.44	0.23	0.23	0.37
Arm span (cm)	0.46	0.54**	0.44	0.42	0.44

* *p* < 0.01.

** *p* < 0.05.

The adequacy of allometric scaling of performance variables in relation to anthropometric variables was tested, specifically with respect to body mass and lean body mass, which showed the strongest correlations with performance in Table 2.

Results indicated that allometric adjustment by body mass was considered adequate only for female participants for the variables 1000-m mean power, maximum aerobic power from the incremental test, and maximal paddling power (Table 3). Therefore, only ergometer-based power variables in females were normalized using their corresponding allometric exponents (b), whereas absolute values were retained for all other comparisons.

**Table 3:** Regression diagnostics and adequacy of allometric scaling of paddling performance variables by body mass in male and female outrigger canoe paddlers.

Variable	Sex	Allometric exponent (b) [95% CI]	R^2^	*p*	Adjustment applied
On-water speed (m.s^−1^)	F	−0.04 [−0.62, 0.54]	<0.01	0.88	No
	M	−0.03 [−0.51, 0.46]	<0.01	0.90	No
1000-m mean speed (m.s^−1^)	F	0.50 [0.16, 0.83]	0.41	<0.01*	No
	M	0.13 [−0.04, 0.29]	0.12	0.12	No
1000-m mean power (W)	F	1.49 [0.47, 2.50]	0.41	<0.01*	Yes
	M	0.39 [−0.11, 0.89]	0.12	0.12	No
Maximal aerobic power (W)	F	1.35 [0.57, 2.13]	0.50	<0.01*	Yes
	M	0.10 [−0.41, 0.61]	<0.01	0.69	No
Maximum paddling power (W)	F	1.31 [0.38, 2.24]	0.41	<0.01*	Yes
	M	0.18 [−0.36, 0.72]	0.03	0.49	No

Allometric scaling was assessed using log–log linear regression models between body mass and each performance variable. R^2^ represents the coefficient of determination of the regression model. Adjustment applied indicates whether the performance variable was normalized using its corresponding allometric exponent (b). F, female; M, male; BM, body mass.

* *p* < 0.05.

Similar results were observed when allometric scaling by lean body mass was applied (Table 4). Allometric adjustment by lean body mass was considered adequate only for female participants for the variables 1000-m mean power, maximal aerobic power from the incremental test, and maximum paddling power. Therefore, only ergometer-based power variables in females were normalized using their corresponding allometric exponents, whereas absolute values were retained for the other variables.

**Table 4:** Regression diagnostics and adequacy of allometric scaling of paddling performance variables by lean body mass in male and female outrigger canoe paddlers.

Variable	Sex	Allometric exponent (b) [95% CI]	R^2^	*p*	Adjustment applied
On-water speed (m.s^−1^)	F	0.16 [−0.43, 0.74]	0.04	0.56	No
	M	−0.11 [−0.58, 0.35]	0.03	0.60	No
1000-m mean speed (m.s^−1^)	F	0.64 [0.39, 0.90]	0.67	<0.01*	No
	M	0.17 [0.01, 0.33]	0.19	0.04	No
1000-m mean power (W)	F	1.93 [1.15, 2,70]	0.67	<0.01*	Yes
	M	0.50 [0.02, 0.99]	0.19	0.04	No
Maximal aerobic power (W)	F	1.64 [1.04, 2.24]	0.71	<0.01*	Yes
	M	0.31 [−0.19, 0.80]	0.08	0.22	No
Maximum paddling power (W)	F	1.68 [0.99, 2.37]	0.68	<0.01*	Yes
	M	0.37 [−0.11, 0.85]	0.12	0.12	No

Allometric scaling was assessed using log–log linear regression models between lean body mass and each performance variable. R^2^ represents the coefficient of determination of the regression model. Adjustment applied indicates whether the performance variable was normalized using its corresponding allometric exponent (b). F, female; M, male.

* *p* < 0.05.

Table 5 presents the simple linear regression models examining the extent to which ergometer-derived performance variables predict on-water paddling performance. In female participants, the 1000-m mean power allometrically adjusted for body mass was the strongest individual predictor of on-water performance (adjusted R^2^ = 0.67, *p* < 0.01; *β* = 7.77), followed by maximum paddling power allometrically adjusted for body mass (adjusted R^2^ = 0.62, *p* < 0.01; *β* = 2.16). In contrast, non-adjusted (absolute) variables and variables adjusted for lean body mass showed weaker or non-significant relationships with on-water performance (adjusted R^2^ ≤ 0.37).

**Table 5:** Simple linear regression analysis of on-water paddling performance (dependent variable) and the ergometer variables (predictors) in male and female outrigger canoeists.

Predictor	R^2^	Adj R^2^	*F* (*p*)	*β* [95% CI]	SEE
Female					
Adjusted Maximum paddling power (W/BM^1.31^)	0.66	0.62	17.07 (<0.01)	2.16 [0.98, 3.35]	0.13
Adjusted Incremental peak power (W/BM^1.35^)	0.53	0.48	10.07 (0.01)	5.83 [1.67, 9.99]	0.15
Adjusted 1000-m mean power (W/BM^1.49^)	0.70	0.67	20.83 (<0.01)	7.77 [3.92, 11.62]	0.12
Adjusted Maximum paddling power (W/LBM^1.68^)	0.40	0.33	5.94 (0.04)	4.36 [0.31, 8.41]	0.17
Adjusted Incremental peak power (W/LBM^1.64^)	0.14	0.04	1.44 (0.26)	5.37 [−4.76, 15.51]	0.20
Adjusted 1000-m mean power (W/LBM^1.93^)	0.43	0.37	6.78 (0.03)	18.38 [2.41, 34.34]	0.16
Maximum paddling power (W)	0.23	0.15	2.75 (0.13)	0.003 [0.00, 0.01]	0.19
Incremental peak power (W)	0.16	0.06	1.66 (0.23)	0.01 [−0.01, 0.02]	0.20
1000-m mean power (W)	0.29	0.21	3.67 (0.09)	0.01 [0.00, 0.02]	0.18
1000-m mean speed (m/s^−1^)	0.32	0.24	4.21 (0.07)	0.47 [−0.05, 0.98]	0.18
Male					
Maximum paddling power (W)	<0.01	−0.09	0.05 (0.83)	−0.0007 [−0.01, 0.01]	0.21
Incremental peak power (W)	0.71	0.69	27.42 (<0.01)	0.01 [<0.01, 0.01]	0.11
1000-m mean power (W)	0.53	0.48	12.27 (<0.01)	0.01 [0.00, 0.01]	0.14
1000-m mean speed (m/s^−1^)	0.53	0.49	12.47 (<0.01)	0.86 [0.32, 1.40]	0.14

W, watts; BM, body mass; LBM, lean body mass. Adjusted variables were normalized using the corresponding allometric exponent derived from log–log regression models.

In male participants, absolute maximum aerobic power from the incremental test explained the largest proportion of variance in on-water performance (adjusted R^2^ = 0.69, *p* < 0.01). The 1000-m mean power and 1000-m mean speed also yielded significant regression models, although with lower explanatory power (adjusted R^2^ = 0.48 and 0.49, respectively; *p* < 0.01).

## 4 Discussion

Due to the popularity of ergometer testing and its importance as a selection, training and monitoring tool used by outrigger canoe clubs, the main aim of the present study was to investigate the relationships between performance in different ergometer tests and anthropometric variables and on-water performance in an OC race simulation in male and female athletes. When paddling performance was analyzed both on ergometer and on-water, the male athletes outperformed the female athletes, presenting faster completion times, higher power outputs, and greater mean speeds across all tests. However, given the significant sex-related differences in anthropometric characteristics, performance test interpretation should consider sex-specific body characteristics, particularly when evaluating ergometer-based assessments in female athletes.

Our findings showed that male paddlers had greater body mass and height compared with female paddlers, consistent with previous studies on rowing (34, 35) and dragon boat athletes (36). Body fat percentage was lower in males than in females in our sample, also in line with findings reported in dragon boat athletes (36). Similarly, studies involving Olympic sprint canoe and kayak paddlers have shown that females athletes present a higher sum of eight skinfolds compared with male athletes (37). In the same way, female rowers have been reported to exhibit lower lean body mass than male rowers (34, 35), which is comparable to the pattern observed in the participants in our study. Taken together, these findings suggest that sex-related differences in body composition remain an important determinant influencing paddling and rowing performance potential. In addition to anthropometric characteristics, the physiological profile of the present sample can be further contextualized by the maximal power output achieved during the incremental test.

The maximum power outputs obtained during the incremental test in the present study (males: 107.9 ± 14.9 W; females: 57.8 ± 12.4 W) were comparable to values previously reported for competitive but non-elite OC paddlers. Humphries et al. (22) reported similar power outputs in competitive regional- and state-level athletes (males: 112.0 ± 19.8 W; females: 65.8 ± 19.8 W) using a modified Concept2 rowing ergometer and an incremental protocol with 15 W increases every minute. Likewise, Kerr et al. (12), who applied a similar incremental protocol with 15 W increases every 2 min on a modified Concept2 ergometer, observed slightly higher power values in trained female paddlers (74 ± 18 W), which may partially reflect their greater competitive experience and more structured training routines.

In contrast, substantially higher maximum power outputs have been reported in elite male paddlers. King et al. (2) documented peak power values of 207.2 ± 38.7 W in elite and 175.4 ± 29.5 W in sub-elite athletes using a different ergometer model (MultiStroke O1-M, Kayak Pro, USA) and an incremental protocol with shorter stage durations (15 W increases every minute). These methodological differences, particularly ergometer design and stage duration, may partly explain the higher power values observed in that study and should be taken into account when interpreting comparisons across studies. Together, these findings suggest that the athletes in this investigation, most of whom had competitive experience ranging from regional to international levels, can be characterized as competitive but non-elite paddlers, with incremental test performances consistent with club- and regional-level standards reported in the literature.

In our study, moderate to strong correlations between ergometer performance and body mass and lean body mass were observed only among female athletes, indicating a positive relationship between body size and ergometer performance in this group. In addition, arm span showed a moderate correlation with performance in the maximum paddling power test in females, suggesting that taller female athletes are able to generate greater power during a 20-stroke maximum paddling power test. Conversely, this variable was not associated with performance in other ergometer tests or on-water performance. Previous studies have suggested that OC athletes with greater sitting height and arm span may benefit from a mechanical advantage (22). Consistently, in kayaking, elite paddlers have been reported to exhibit greater height and sitting height compared with non-selected athletes (17). In rowing, greater stature has also been associated with superior performance (34), potentially due to longer stroke length (38), which is a key determinant of rowing performance (13). Nevertheless, in the present study, neither the height nor sitting height correlated significantly with performance in any test, indicating that, regardless of sex, these anthropometric characteristics were not essential determinants of paddling performance. Although taller athletes generally present greater arm span and sitting height, which may favor longer stroke lengths, athletes with smaller body size may compensate for this disadvantage by increasing stroke rate, using greater trunk motion, and relying on isometric leg contractions, as suggested by Kerr et al. -(24) in female paddlers. In the present sample, male athletes were taller and had greater arm span than females; although, mean stroke rate during the 1000-m time-trial did not differ significantly between sexes. This suggests that the compensatory strategy of increasing stroke rate to offset smaller body size was not adopted by female athletes in this study.

Previous studies have suggested that although greater body mass may enhance absolute power output on an ergometer, excessive body mass can impair on-water performance by increasing hydrodynamic drag (26, 27). In the current analysis, different patterns were observed between males and females when focusing on ergometer-based tests. Among male athletes, no significant correlations were found between body mass and performance in either ergometer or on-water tests. In contrast, female athletes exhibited statistically significant moderate to strong correlations between body mass and ergometer performance across all three tests (incremental test, 1000-m time-trial, and maximum paddling power test) but no significant relationship between body mass and on-water performance. These findings suggest that body mass influences ergometer performance in females but does not directly translate to on-water outcomes, highlighting modality-specific determinants of paddling performance. To date, only one study has examined the relationship between body mass and on-water performance in OC athletes, reporting a moderate negative correlation (r = −0.51) between 500-m sprint time in the V1 boat and body mass (2). This negative coefficient indicates that athletes with greater body mass tended to complete the sprint in a shorter time, suggesting a performance advantage in this short-distance event. In addition, stronger correlations were observed in females when total lean body mass was considered in relation to ergometer performance, indicating that athletes with greater lean body mass achieved superior power outputs on the ergometer. However, similar to body mass, total lean body mass was not significantly associated with on-water performance in either females or males.

To further investigate the role of anthropometric characteristics in ergometer performance, allometric scaling analyses were conducted for body mass and total lean body mass in female and male athletes. This approach has been shown to improve the interpretation of rowing performance by accounting for the mathematical influence of body size on power output (28, 29). In the present sample, significant allometric relationships were observed only among female athletes, and exclusively for power-based ergometer variables. The allometric exponents for these measures ranged from 1.31 to 1.49, indicating a strong dependence of ergometer-derived power on body mass in this group. Similarly, when the total lean body mass was considered, significant allometric relationships were also observed only in females, with exponents ranging from 1.64 to 1.93 for power-based ergometer variables. No significant allometric associations were identified for male athletes or for paddling speed variables, either on the ergometer or on water, in either sex. Based on these findings, only ergometer-derived power variables for female athletes were normalized using their respective allometric exponents.

The allometric exponents obtained for female athletes indicate that ergometer-derived power increased disproportionately with body mass and total lean body mass. Physiologically, this pattern suggests that heavier and leaner athletes benefit from a mechanical advantage in force production, as greater body mass and lean body mass are often accompanied by higher absolute muscle mass and greater propulsive leverage (39). As a result, heavier paddlers are able to generate power at a rate that increases more than proportionally with their body size. However, this mass-dependent advantage appears to be specific to ergometer conditions, where body weight does not impose a hydrodynamic penalty (21). In the present study, neither body mass nor total lean body mass was significantly associated with on-water performance. This finding contrasts with the notion that on-water paddling is constrained by drag forces, which increase with boat displacement and wetted surface area. Such constraints may limit the transfer of mass-related gains in power to actual paddling speed. The high allometric exponents observed in female athletes therefore reinforce the idea that ergometer power is strongly influenced by body size, but that this mechanical advantage does not necessarily translate into superior on-water performance. In ergometer rowing, larger and heavier athletes typically outperform lighter athletes when technical skill is comparable. However, excess body weight in the boat tends to impair on-water performance (28). Interestingly, this ergometer-related difference is less evidenced on the water. For example, the mean performance difference between heavyweight and lightweight male rowers has been reported to be approximately 9 s over an average race time of 354 s. This corresponds to a performance decrement of only about 2.5% for lightweight athletes (26). This reduced performance gap between weight categories in on-water conditions suggests that body mass plays a different role in aquatic locomotion compared with ergometer performance. Although larger athletes are generally capable of producing greater absolute power, real-world performance ultimately depends on achieving an effective power-to-mass ratio, which is particularly relevant for movement efficiency in endurance-based activities such as road cycling and, especially, rowing on water (28).

To our knowledge, this is the first study to examine the relationship between laboratory-based performance tests and actual race performance in OC. This represents a novel contribution to the literature, as most existing evidence on paddling physiology derives from Olympic rowing, canoeing or kayaking (5, 7, 17, 27). In one of the earliest and most comprehensive analyses in kayaking, Fry and Morton (17) demonstrated that both anaerobic and aerobic parameters contribute meaningfully to performance across all race distances. Unlike running, where a 100-m sprint lasts about 10 s, elite 500-m kayak sprint races last around 105 s, indicating substantial aerobic involvement even in relatively short events. A similar pattern is expected in OC, in which elite paddlers complete 500-m races in approximately 2 min. Fry and Morton (17) further showed that, as race distance increases (500 m, 1000 m, 10 km, 42 km), the contribution of aerobic parameters becomes progressively more important. In their study, time to exhaustion during an incremental test was the strongest predictor of performance in the 42-km event (*r* = – 0.843). This finding highlights the critical role of aerobic power and sustainable force production. This pattern aligns with the present findings in male athletes, for whom incremental peak power was the strongest predictor of on-water performance (*r* = 0.69), followed by 1000-m mean speed and power (*r* = 0.49 and 0.48, respectively). In contrast, the 20-strokes maximum paddling power test did not correlate significantly with on-water performance in males, suggesting that this short-duration test did not meaningfully influence race outcomes in this group. Among female paddlers, however, the strongest predictor of on-water performance was 1000-m mean power allometrically adjusted for body mass (*r* = 0.67), followed by body-mass adjusted maximum paddling power and adjusted incremental peak power (*r* = 0.62 and 0.48, respectively). These findings indicate that performance in both longer and shorter ergometer-based tests is relevant for predicting on-water performance in female athletes when body mass is appropriately accounted for. Collectively, these sex-specific differences underscore the importance of considering body size, particularly in female athletes, when interpreting ergometer-derived performance measures in relation to real-world OC racing outcomes.

This study presented some methodological limitations related to the equipment used during testing. First, all athletes used the same paddle length during ergometer testing. Although this standardized approach was necessary due to equipment availability logistical constraints, it may have favored athletes with specific anthropometric characteristics, particularly taller paddlers, and may have influenced stroke mechanics and technique in athletes with smaller body size. Second, although all athletes competed using OC1 (V1R) boats, the specific canoe model, manufacturer, and boat weight were not standardized. In line with this, paddle characteristics were individualized, as each paddler used their own paddle, reflecting typical competition settings. Variations in canoe construction, materials, and weight may influence hydrodynamic efficiency and paddling mechanics, potentially contributing to unexplained variance in on-water performance. Despite this, this design was intentionally adopted to preserve ecological validity, as canoe model and paddle specifications are generally not standardized in official outrigger canoe competitions. To minimize environmental variability, all athletes competed simultaneously within sex-specific heats, thereby reducing potential environmental confounding effects (e.g., wind, water conditions, and tide fluctuations).

Beyond equipment-related considerations, statistical limitations should also be acknowledged. The relatively smaller female sample size may limit generalizability and increase uncertainty in the regression estimates. Even so, only simple linear regression models with a single predictor were applied, which minimized model complexity and reduced the likelihood of overfitting. Future studies including larger female cohorts are warranted to confirm the observed associations and further explore potential sex-specific predictors of performance.

The results of this study demonstrate that male paddlers exhibited superior absolute performance across all ergometer and on-water tests. In contrast, female athletes showed a stronger dependence of ergometer-derived power on body mass and total lean body mass, as evidenced by significant allometric relationships. These findings suggests that heavier and leaner female paddlers achieve superior performance under ergometer conditions. Among male athletes, raw incremental peak power was the strongest predictor of on-water performance, whereas in females, the 1000-m mean power allometrically adjusted for body mass provided the best prediction of on-water performance. Overall, these findings highlight the importance of considering both physiological and anthropometric factors when interpreting ergometer-based assessments and their relationship with real-world on-water performance.

## Data Availability

The raw data supporting the conclusions of this article will be made available by the authors, without undue reservation.
